# Clusters of clinical and immunologic features in patients with bullous systemic lupus erythematosus: experience from a single-center cohort study in China

**DOI:** 10.1186/s13023-022-02445-z

**Published:** 2022-07-23

**Authors:** Lin Qiao, Bingjie Zhang, Wenjie Zheng, Mengtao Li, Yan Zhao, Xiaofeng Zeng, Fengchun Zhang, Li Wang, Li Li

**Affiliations:** 1 Department of Rheumatology and Clinical Immunology, Chinese Academy of Medical Sciences & Peking Union Medical College; National Clinical Research Center for Dermatologic and Immunologic Disease (NCRC-DID), Ministry of Science & Technology; State Key Laboratory of Complex Severe and Rare Diseases, Peking Union Medical College Hospital (PUMCH); Key Laboratory of Rheumatology and Clinical Immunology, Ministry of Education, Beijing, China; 2 Department of Dermatology, State Key Laboratory of Complex Severe and Rare Diseases, Peking Union Medical College Hospital, Chinese Academy of Medical Science and Peking Union Medical College, National Clinical Research Center for Dermatologic and Immunologic Diseases, Beijing, 100730 China

**Keywords:** Bullous systemic lupus erythematosus, Blisters, Vesiculobullous, Active SLE

## Abstract

**Background:**

Bullous systemic lupus erythematosus (BSLE) is a rare subtype of systemic lupus erythematosus (SLE) that is clinically characterized by subepidermal tense vesicles or bullae. We aimed to investigate the clinical and laboratory features of patients with BSLE.

**Methods:**

We retrospectively reviewed all patients who fulfilled the diagnostic criteria for BSLE in our institution from 2015 to 2021. Cutaneous lesions, systemic manifestations, treatment options, and outcomes were evaluated. For each case of BSLE, four controls were randomly selected from patients with single SLE. Major clinical and laboratory characteristics were compared between the two groups.

**Results:**

Among 4221 patients with SLE, 12 developed BSLE. Vesiculobullous lesions were the first sign in five of the BSLE patients (5/12, 41.7%) and appeared after SLE diagnosis in the remaining seven patients (7/12, 58.3%), with a median duration from SLE onset of 36 months (4–115 months). The most common BSLE-affected sites were the head and neck (10/12, 83.3%), extremities (9/12, 75.0%), trunk (7/12, 58.3%), and mucosae (6/12, 50.0%). All patients with BSLE had extra-cutaneous involvement. The SLE disease activity index score exceeded 5 in 10/12 (83.3%) patients, which indicated high disease activity. Patients in the BSLE group had significantly higher incidences of proteinuria (83.3% vs. 47.9%, *P* = 0.027), hematuria (75% vs. 31.3%, *P* = 0.006), hemolytic anemia (33.3% vs. 0%, *P* = 0.000), and leukopenia (66.7% vs. 25.0%, *P* = 0.006) than those in the control group. The use of systemic corticosteroids, immunosuppressants, dapsone, and skin care was effective in controlling disease.

**Conclusions:**

Vesiculobullous lesions may be the first manifestation and indicate a high disease activity in patients with BSLE. Early diagnosis using clinical, histopathological, and immunological evaluations can lead to appropriate treatment of this progressive disease and improve prognosis.

## Significance and innovations

- BSLE is a distinct form of SLE described mainly in case reports and case series. Here we present a case series on BSLE in a single center in China.
- Vesiculobullous lesions may be the first manifestation and indicate a high disease activity in patients with BSLE.

## Introduction

Systemic lupus erythematosus (SLE) is a chronic autoimmune disease that can affect any organ. Cutaneous involvement, observed in 72%–85% of patients with SLE, is a common feature with a tremendous variability during the course of the disease [1, 2]. Bullous SLE (BSLE) is clinically characterized by tense subepidermal vesicles or bullae, and remains an uncommon subtype of SLE, which is rarely described in large case series [3–5].

The diagnostic criteria for BSLE were first described by Camisa and Sharma in 1983 [6] and revised in 1988 [7]. These criteria included: (1) a diagnosis of SLE based on the American College of Rheumatology (ACR) criteria; (2) the presence of vesicles and/or bullae; (3) the presence of histopathological features similar to those of dermatitis herpetiformis (DH); (4) direct immunofluorescence (DIF) findings showing IgG, IgM, or IgA at the basement membrane zone (BMZ); and (5) indirect immunofluorescence (IIF) (performed using the salt-split skin technique) findings negative or positive for circulating autoantibodies against the BMZ. In 1995, Yell et al. defined BSLE as "an acquired subepidermal blistering disease in a patient with SLE, with immune reactants at the BMZ on either DIF or IIF" [8]. Moreover, they highlighted three types of BSLE. Type I BSLE, the most common type, requires the presence of autoantibodies to type VII collagen, either circulating or deposited as determined via IIF or direct immunoelectron microscopy, respectively. Type II BSLE is characterized by the absence of autoantibodies to type VII collagen. Type III BSLE, the most recently proposed type, requires the presence of autoantibodies that bind either epidermal or both dermal and epidermal epitopes.

We therefore conducted a retrospective study to describe the clinical, immunological and histological presentations of BSLE, and compare clinical and laboratory features between BSLE patients and single SLE group. The aim was to help clinicians better understand this rare disease and prevent disease progression.

## Patients and methods

### Patients

We retrospectively reviewed inpatients admitted in Peking Union Medical College Hospital between January 2015 and December 2021. All patients were diagnosed with SLE based on the 2012 Systemic Lupus Erythematosus International Collaborating Clinics classification criteria or the 2019 European League against Rheumatism/ACR classification criteria [9, 10] and the diagnosis was confirmed by at least two rheumatologists. Furthermore, at least two dermatologists confirmed the diagnosis of BSLE.

For each case of BSLE, four sex-and age-matched controls were randomly and contemporaneously selected from single SLE patients. This study was approved by the Medical Ethics Committee of Peking Union Medical College Hospital (approval number: S-K1585). All the subjects provided written informed consent prior to inclusion in the study.

### Statistical analyses

Data analysis was performed using SPSS version 26.0 (SPSS Inc., Chicago, IL, USA). Numerical and categorical data were expressed as mean ± SD (range) and percentage, respectively. The level of significance was estimated using the Student’s *t*-test, Pearson’s chi-square test, or Fisher’s exact test (when expected frequencies were < 5). Statistical significance was set at *P* < 0.05.

## Results

### Demographic characteristics

We reviewed 4221 patients and identified 12 patients with BSLE, 10 (83.3%) of whom were women. The mean age was 25.9 ± 13.3 years (range 7–56 years), and the median disease duration was 17 months (range 3–43 months). Vesiculobullous lesions occurred as an initial manifestation in 5 (41.7%) patients and after SLE diagnosis in the remaining 7 (58.3%) patients, with a median duration from onset of 36 months (range 4–115 months).

### Clinical characteristics of skin lesions

**Fig. 1:**
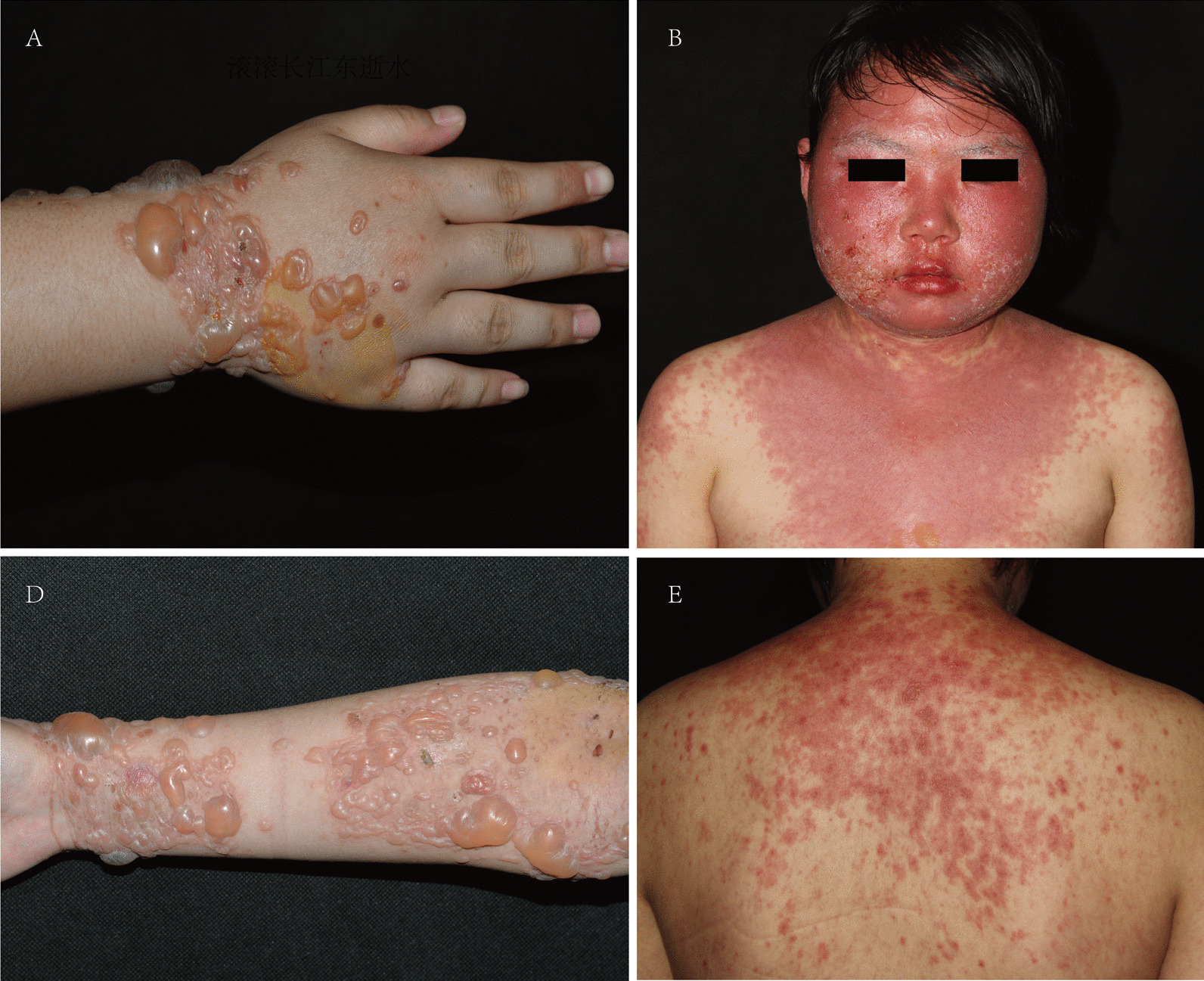
Clinical pictures of patients with tense bullae and edematous erythematous plaques located in the face, trunk, and extremities

**Fig. 2:**
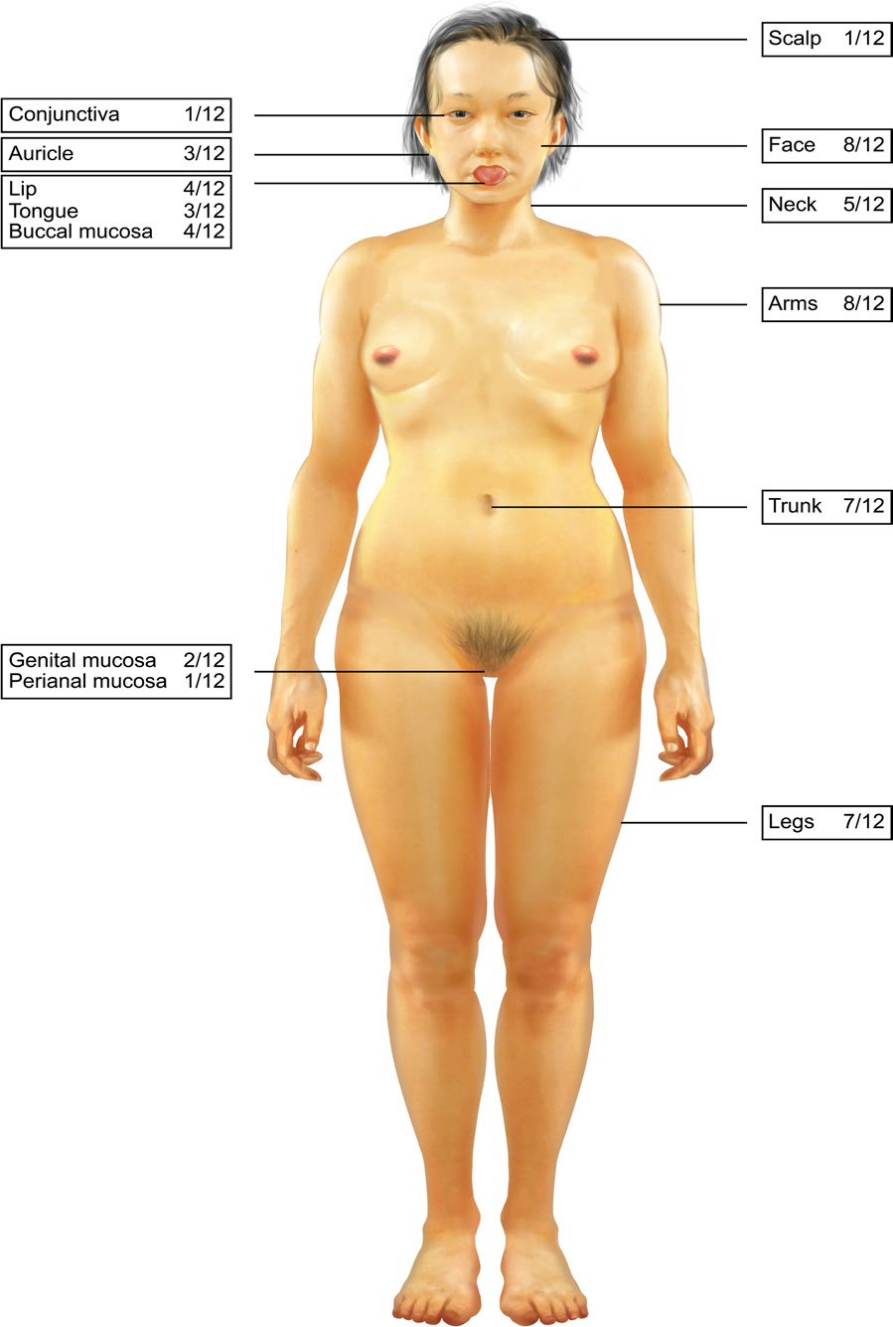
The distribution of skin lesions

The cutaneous characteristics of BSLE are tense vesicles and bullae involving the face, trunk, extremities and mucosae, mostly accompanied by pruritus and pain. Bloody blisters may also occur. Edematous erythematous plaques were observed in 9 of 12 patients (9/12, 75.0%) (Fig. 1). Skin lesions were distributed over the head and neck (10/12, 83.3%), extremities (9/12, 75.0%), trunk (7/12, 58.3%), and mucosae (6/12, 50.0%). Mucosal involvement included the buccal mucosa (4/12, 33.3%), lip (4/12, 33.3%), tongue (3/12, 25.0%), auricle (3/12, 25.0%), genital mucosa (2/12, 16.7%), perianal mucosa (1/12, 8.3%), and conjunctiva (1/12, 8.3%) (Fig. 2).

**Fig. 3:**
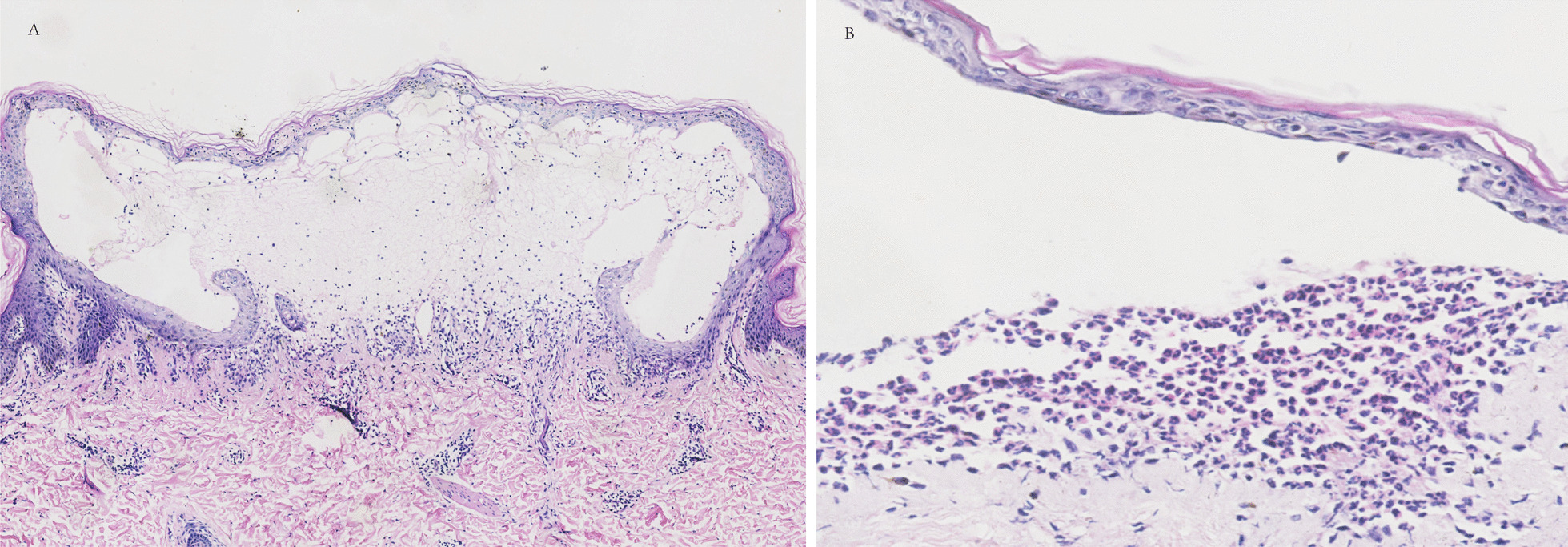
Skin biopsy showing subepidermal blisters (**A**) and dense neutrophilic infiltration in the blister (**B**) (hematoxylin–eosin magnifications, A × 100, B × 200)

Skin biopsy from 10 of the 12 patients with BSLE revealed similar histopathological features. The epidermis was usually uninvolved. Subepidermal blisters, the most representative features, appeared in nearly all skin biopsy findings (8/10, 80%), with diffuse monotonous neutrophilic infiltration in the blister (7/10, 70%). Perivascular dermal infiltrates were mainly composed of neutrophils and nuclear dust (7/10, 70%), and sometimes mixed inflammatory cells (3/10, 30%) (Fig. 3). Anti-BMZ antibodies were found in 66.7% (8/12) of the cases via IIF and the binding was located on the dermal side in all these patients via IIF on split skin. DIF was positive at the BMZ in 4 of 8 BSLE patients, with a linear deposition of mixed immune complexes including IgG (4/8, 50%), IgM (4/8, 50%), IgA (2/8, 25%), and C3 (1/8, 12.5%) (Table 1). In addition, serum anti-BP180 and anti-BP230 antibodies were negative in all patients on performing ELISA. Anti-type VII collagen antibody was positive in 66.7% of the patients (Table 2).

**Table 1:** Demographic and clinical characteristics of the patients with BSLE

Parameter	Value
Age at BSLE diagnosis (years, mean ± SD)	25.9 ± 13.3
Sex	
Female	10/12 (83.3%)
Male	2/12 (16.7%)
BSLE as an initial feature of SLE	5/12 (41.7%)
Distribution of vesiculobullous lesions	
Head and neck	10/12 (83.3%)
Extremities	9/12 (75.0%)
Trunk	7/12 (58.3%)
Mucosal involvment	6/12 (50.0%)
Buccal mucosa	4/12 (33.3%)
Lip	4/12 (33.3%)
Tongue	3/12 (25.0%)
Auricle	3/12 (25.0%)
Genital mucosa	2/12 (16.7%)
Perianal	1/12 (8.3%)
Conjunctiva	1/12 (8.3%)
Histological features	
Subepidermal blister	8/10 (80.0%)
Neutrophil infiltrate in blister	7/10 (70.0%)
Neutrophil infiltrate in dermis	7/10 (70.0%)
Polymorphous infiltrate in dermis	3/10 (30.0%)
DIF at the BMZ	4/8 (50.0%)
Linear deposition	4/8 (50.0%)
IgG deposition	4/8 (50.0%)
IgM deposition	4/8 (50.0%)
IgA deposition	2/8 (25.0%)
C3 deposition	1/8 (12.5%)

*DIF* Direct immunofluorescence, *BMZ* Basement membrane zone

**Table 2:** Immunofluorescence, systemic findings, treatment, and outcome of the patients with BSLE

No	Age/Sex	IIF	DIF	ELISA			Systemic findings	SLEDAI	SLE characteristic laboratory values	Treatment	Outcome
				Anti-BP180 antibody	Anti-BP230 antibody	Anti-type VII collagen antibody					
1	M/36	Anti-BMZ antibodies located on the dermal side	No data	Negative	Negative	Negative	AIHA, leukopenia, arthritis, LN, NPSLE, serositis	21	Hypocomplementemia, ANA H1:320, positive anti-dsDNA and anti-rRNP	GC (pulse), CTX, MMF	Lost
2	F/17	Anti-BMZ antibodies located on the dermal side	Negative	Negative	Negative	Positive	LN	4	ANA HS1:80, positive anti-rRNP, anti-Sm and anti-SSA	GC, CsA, HCQ	Lost
3	F/13	Anti-BMZ antibodies located on the dermal side	linear IgG and IgM on BMZ	Negative	Negative	Positive	LN, hemocytopenia	10	Hypocomplementemia, ANA H:1280, positive anti-dsDNA and anti-rRNP	GC, CTX, MMF, HCQ	Improved
4	M/32	Negative	Negative	Negative	Negative	Positive	LN, hemocytopenia, PAH, serositis	25	Hypocomplementemia, ANA H:1280, positive anti-dsDNA	GC (pulse), CTX	Improved
5	F/24	Anti-BMZ antibodies located on the dermal side	No data	Negative	Negative	Positive	LN, hemocytopenia, myocarditis, NPSLE, serositis	19	Hypocomplementemia, ANA S1:80, positive anti-RNP	GC (pulse), HCQ	Lost
6	F/13	Anti-BMZ antibodies located on the dermal side	Linear IgG and IgM on BMZ	Negative	Negative	Positive	LN, hemocytopenia, arthritis, serositis	3	Hypocomplementemia, ANA H1:640, positive anti-dsDNA, anti-SSA and anti-SSB	GC (pulse), CTX	Relapsed
7	F/36	Negative	Linear IgG, IgM, IgA and C3 on BMZ	Negative	Negative	Negative	Arthritis, LN, ILD	6	ANA S1:1280, positive anti-RNP, anti-SSA and anti-SSB	GC (pulse), CTX, HCQ	Improved
8	F/27	Negative	Negative	Negative	Negative	Positive	LN, IPO, hemocytopenia	10	Hypocomplementemia, ANA H1:320, positive anti-dsDNA, anti-SSA and anti-SSB	GC (pulse*2), CTX, FK506, HCQ	Improved
9	F/28	Anti-BMZ antibodies located on the dermal side	No data	Negative	Negative	Negative	LN, hemocytopenia, arthritis, serositis	20	Hypocomplementemia, ANA H1:160, positive anti-dsDNA, ACL, and LA	GC (pulse), CTX, HCQ	Improved
10	F/22	Anti-BMZ antibodies located on the dermal side	No data	Negative	Negative	Positive	LN, serositis	12	Hypocomplementemia, ANA HS1:320, positive anti-dsDNA, anti-Sm, anti-RNP and anti-SSA	GC, MMF, HCQ	Improved
11	F/56	Negative	Negative	Negative	Negative	Positive	Hemocytopenia, arthritis,	8	Hypocomplementemia, ANA S1:160, positive anti-SSA	GC, TGP	Relapsed
12	F/7	Anti-BMZ antibodies located on the dermal side	Linear IgG, IgM, and IgA on BMZ	Negative	Negative	Negative	Hemocytopenia, arthritis, LN, ILD, NPSLE	18	Hypocomplementemia, ANA cytoplasmic 1:320, positive anti-dsDNA and anti-rRNP	GC, FK506, HCQ	Improved

*IIF* indirect immunofluorescence, *DIF* direct immunofluorescence, *BMZ* basement membrane zone, *ELISA* enzyme-linked immunosorbent assay, *SLEDAI* SLE disease activity index, AIHA: autoimmune hemolytic anemia, *LN* lupus nephritis, *NPSLE* neuropsychiatric systemic lupus erythematosus, *IPO* intestinal pseudo-obstruction, *ILD* interstitial lung disease, *GC* glucocorticoid, *CTX* cyclophosphamide, *MMF* mycophenolate mofetil, *HCQ* hydroxychloroquine, *TGP* total glucosides of peony, FK506 tacrolimus, *CsA* cyclosporine A, *Anti-dsDNA* anti-double stranded DNA antibody, *Anti-Sm* anti-Smith antibody, *Anti-SSA* anti-SSA antibody, *Anti-SSB* anti-SSB antibody, *Anti-RNP* anti-u1 small-nuclear RNA–protein antibody, *Anti-rRNP* antiribosomal RNA–protein antibody, *LA* lupus anticoagulant, *ACL* anti cardiolipin antibody

### Systemic manifestations of SLE

All patients with BSLE had extra-cutaneous organ involvements, including lupus nephritis (11/12, 91.7%), hemocytopenia (10/12, 83.3%), alopecia (7/12, 58.3%), fever (7/12, 58.3%), serositis (6/12, 50.0%), arthritis (6/12, 50.0%), neurological involvement (3/12, 25.0%), cardiac dysfunction (2/12, 16.7%), Raynaud phenomenon (1/12, 8.3%), interstitial lung disease (1/12, 8.3%), and gastrointestinal involvement (1/12, 8.3%). Further, four patients had concurrent Sjogren’s syndrome. The mean SLE disease activity index (SLEDAI) score of patients with BSLE upon admission was 13.0 ± 7.3 and SLEDAI scores exceeded 5 in 10/12 (83.3%) patients, indicating high disease activity. Ten patients had hypocomplementemia. The levels of complement C3 and C4 were 0.58 ± 0.34 g/L and 0.09 ± 0.08 g/L, respectively (Table 2).

### Comparison between BSLE and single SLE patients

The incidences of proteinuria (83.3% vs. 47.9%, *P* = 0.027), hematuria (75% vs. 31.3%, *P* = 0.006), hemolytic anemia (33.3% vs. 0%, *P* = 0.000), and leukopenia (66.7% vs. 25.0%, *P* = 0.006) were significantly higher in patients with BSLE than in the single SLE group (Table 3).

**Table 3:** Major clinical and laboratory characteristic of BSLE and control SLE group

Variable	BSLE group (n = 12)	Single SLE group (n = 48)	*P* value
Age (years, mean ± SD)	25.9 ± 13.3	27.3 ± 14.4	0.766
Gender (F/M)	2/10	4/44	0.389
Disease duration (months, mean ± SD)	26.5 ± 32.9	46.0 ± 51.2	0.215
Arthritis (n, %)	6 (50.0)	22 (45.8)	0.796
Renal involvement (n, %)	11 (91.7)	25 (52.1)	0.012*
Nephrotic syndrome (n, %)	4 (33.3)	6 (12.5)	0.083
Proteinuria (n, %)	10 (83.3)	23 (47.9)	0.027*
Hematuria (n, %)	9 (75.0)	15 (31.3)	0.006*
Renal insufficiency (n, %)	4 (33.3)	6 (12.5)	0.083
Hemocytopenia (n, %)	10 (83.3)	19 (39.6)	0.007*
Hemolytic anemia (n, %)	4 (33.3)	0 (0.0)	0.000*
Leukopenia (n, %)	8 (66.7)	12 (25.0)	0.006*
Thrombocytopenia (n, %)	2 (16.7)	12 (25.0)	0.542
Myositis (n, %)	0 (0)	3 (6.3)	0.374
Neurological involvement (n, %)	3 (25.0)	9 (18.8)	0.628
Cardiac damage (n, %)	2 (16.7)	17 (35.4)	0.212
Gastrointestinal involvement (n, %)	1 (8.3)	8 (25.0)	0.449
Anti-dsDNA antibody (n, %)	8 (66.7)	23 (47.9)	0.245
Anti-Sm antibody (n, %)	2 (16.7)	7 (14.6)	0.857
Anti-RNP antibody (n, %)	2 (25.0)	14 (29.2)	0.774
Anti-rRNP antibody (n, %)	4 (33.3)	6 (12.5)	0.083
Anti-SSA antibody (n, %)	6 (50.0)	16 (33.3)	0.284
Anti-SSB antibody (n, %)	3 (25.0)	3 (6.3)	0.053

*Denotes *P* < 0.05

### Treatment and prognosis

During the remission induction therapy, all patients received systemic glucocorticoids (GCs). Seven patients underwent GC pulse therapy (methylprednisolone 0.5–1 g/day for 3 days) and one of the seven patients underwent two sessions of GC pulse therapy. Ten patients used immunosuppressants, eight of whom underwent GC-immunosuppressant combination therapy. Cyclophosphamide (CTX) was the most frequently used immunosuppressant (7/12, 58.3%), followed by mycophenolate mofetil (MMF) (3/12, 25.0%), tacrolimus (FK506) (2/12, 16.7%), and cyclosporine A (CsA) (1/12, 8.3%). Moreover, eight patients received hydroxychloroquine (HCQ) in combination with immunosuppressants (8/12, 66.7%). Intravenous gamma globulin therapy was initiated in four patients. Dapsone was only used in one patient because it was difficult to obtain. In addition, skin care was essential in the treatment of skin lesions. Daily rupturing of tense blisters was performed and high-potency topical corticosteroids were used. Notably, patients in our research improved slowly, with a mean hospitalization duration of 32 days. On discharge, the mean SLEDAI score of patients with BSLE was 5.3 ± 3.4.

Nine patients were followed up regularly, with a median follow-up duration of 53 months. All of them received a maintenance dose of prednisolone of < 0.5 mg/kg/day (15 mg and < 15 mg in 1 and 8 patients, respectively). Seven patients received immunosuppressants in addition to prednisolone. The most frequently used immunosuppressive agents were MMF (5/9, 55.6%), FK506 (2/9, 22.3%), thalidomide (1/9, 11.1%), azathioprine (AZA, 1/9, 11.1%), and total glucosides of peony (1/9, 11.1%). Seven patients (7/9, 77.8%) received HCQ. During the follow-up period, 7 of 9 patients experienced complete resolution of skin lesions and pruritus. Only 2 patients (2/9, 22.2%) showed relapse during maintenance therapy, who presented with new-onset erythema and blisters after achieving disease control.

## Discussion

BSLE is a rare autoimmune subepidermal blistering disease that occurs in patients with SLE [11]. Although bullous lesions mostly develop after preexisting SLE, several studies report BSLE may be the first clinical manifestation of SLE. In our research, vesiculobullous lesions occurred as an initial sign in five BSLE patients (5/12, 41.7%) and after SLE diagnosis in the remaining individuals (7/12, 58.3%), with a mean duration from SLE diagnosis of 40.1 ± 36.0 months (range 4–115 months), which was in line with previous studies. Tense blisters mostly appeared on the surface of erythema, rather than normal skin (75% *vs.* 25%). In addition, blisters and erosions could be distributed widely all over the body, including mucosal sites, which has been previously confirmed [12].

The pathogenesis of BSLE is likely related to the presence of autoantibodies to type VII collagen, which is an anchoring fibril that attaches the dermis to the epidermis. Circulating antibodies that target type VII collagen cause complement-mediated leukocyte recruitment and basement membrane-dermal adhesion weakening [13, 14]. Anti-type VII collagen antibody levels are reportedly correlated with disease activity [5]. According to the classification of subtypes in BSLE [15], eight patients were classified as having Type I BSLE due to the presence of autoantibodies reacting with collagen VII and the rest four patients were classified as having Type II BSLE.

Many subepidermal blistering disorders, including bullous pemphigoid (BP), DH, Linear IgA bullous dermatosis (LABD) and epidermolysis bullosa acquisita (EBA), share similar clinical features with BSLE. Thus, diagnostic tests are essential in differentiating these conditions, especially when patients present with tense vesicles and blisters. In our study, skin biopsies of patients with BSLE were characterized by subepidermal blisters and dense neutrophilic infiltration in the upper dermis and bullae, which were consistent with previous literature [12]. Interestingly, the analysis of DIF demonstrated that the type of immune complexes deposited on BMZ is different from that in the previous literature [3], especially the proportion of C3 deposition (12.5% *vs.* 67%). A large retrospective cohort study of BP showed that C3 deposition was associated with the detection of anti-BP180 NC16A autoantibodies and the presence of neutrophils in skin lesions [16]. We speculate that C3 deposition could also influence the immunological and histological features of BSLE. Unfortunately, four patients in our study had no data on DIF; two of them had undergone DIF, but the data was lost as DIF was performed a long time prior. The other two patients did not undergo DIF because they did not present blisters and erosions when they first visited the dermatology department. Further studies with larger groups of patients are needed for confirmation. Additionally, the positive rates of serological tests, including indirect IF on split skin and anti-type VII collagen antibody by ELISA, were generally consistent with those reported in the literature [3]. In our study, serum anti-BP180 and anti-BP230 antibodies were negative in all patients, which contributed to exclude the diagnosis of BP. However, some studies found that a small number of patients with BSLE showed positive for anti-BP180 and anti-BP230 antibodies due to an epitope spreading immune phenomenon [3, 17]. Therefore, the diagnosis of BSLE needs to combine the results of histopathology, DIF, IIF, and serologic tests.

The common type of systemic involvement in BSLE is still controversial. In a French cohort, BSLE was usually accompanied with cytopenia, arthritis, and lupus nephritis [3]. Another retrospective review of patients with BSLE suggested that hematologic and renal involvements were the most frequently associated systemic abnormalities, followed by arthritis [18]. In our study, all patients with BSLE had systemic involvement, such as renal and hematologic involvement, as well as arthritis. Specifically, patients with BSLE had a high frequency of proteinuria, hematuria, hemolytic anemia, and leukopenia. Moreover, 83% of patients with BSLE had a high disease activity (SLEDAI score ≥ 5). However, there was no significant difference in the SLEDAI scores between the BSLE and single SLE groups. Large-scale clinical studies are warranted to confirm the connection between BSLE and disease activity of SLE.

The current therapeutic regimens for BSLE still lacks large scale investigations. Based on clinical experience, dapsone is suggested as an effective treatment in controlling bullous lesions. However, it is usually not the first choice in BSLE because it has little benefit on systemic complications and high incidence (23%) of side effects (hemolysis, hepatic toxicity, and renal toxicity) [19]. If there is severe systemic involvement, the patients should be treated first with systemic GCs as well as immunosuppressants including CTX, CsA, AZA, MMF, and MTX [19, 20]. In our study, some patients accepted the treatment of total glycoside of paeony, which is a traditional Chinese medicine that has anti-inflammatory and immune regulatory effects, and is widely used for the treatment of SLE [21]. Less frequently, the use of anakinra, intravenous immunoglobulins, and rituximab has been reported in patients with refractory disease [3]. In addition, skin care and topical treatment is also essential. Daily rupturing of tense blisters, leaving the blister roof in place, and application of high-potency topical corticosteroids is recommended based on clinical experience. Significantly, infection is a potential risk in patients with BSLE, particularly when extensive skin erosions are present and systemic immunosuppressants are being used for treatment. As a rare and severe condition in patients with SLE, BSLE management requires multidisciplinary cooperation, including rheumatologists, dermatologists, and nurses. Practitioners should focus on promoting epidermal healing and avoiding an infection of the exposed and affected skin. In our study, all patients with BSLE achieved disease control after treatment, and only 22.2% of them relapsed during maintenance therapy, which seemed to be lower than that in previous studies [4, 22].

## Conclusions

Skin lesions may be the first manifestation and indicate a high disease activity in patients with BSLE. Therefore, careful clinical, immunological, and histopathologic evaluations, as well as rigorous treatment options are necessary in patients who present with tense vesicles and/or bullae, especially with concomitant multi-organ involvement. The cooperation of rheumatologists, dermatologists, and nurses plays a key role in early diagnosis and effective treatment.

## Data Availability

Data are available for collaborative studies with qualified investigators after inquiry.
